# Micro-Organisms of Dental Caries

**Published:** 1889-11

**Authors:** 


### Micro-Organisms of Dental Caries.

(Biological Association of Paris, Meeting of March 16, 1889.)

Messrs. Galippe and Vignal have studied the microbes which penetrate into the little dentine canals, and which alone play an effective part in the destruction of teeth. They cleansed the dental cavity, crushed the tooth, enveloped in sterilized paper, in a vice, and sowed the several particles in different media. In this way they obtained six varieties of micro-organisms.

1. Small, short and thick bacillus is found constantly, coagulates milk with formation of lactic acid.

2. Bacillus twice as long as broad, slightly narrowed in the middle, also forms lactic acid in milk.

3. Bacillus, similar to the preceding one, without narrowing, forms long chains, does not coagulate milk, prevents caseine from coagulating with acids, and transforms milk into a yellowish brown fluid.

4. Very short and tender bacillus, about as long as broad, resembling a coccus. Transforms caseine, which soon exhales an unpleasant odor and takes a brown coloration, such as the media of culture. It dissolves fibrine.

5. Roundish bacillus 4-5 fl long, found eight times only. It transforms milk without coagulating it into a brown fluid, which after some time becomes black and exhales a bad odor.

6. Coccus, comparatively voluminous 6 fl in diameter, found five times only in teeth of advanced cariousness with wide canaiculi. It coagulates milk and forms lactic acid.

In addition, the pulp furnished the following three varieties :

1. Bacterium termo, which is met with in all albuminous substances in a state of decomposition.

2. Acts in the same way on albuminous bodies, interverts sugar and forms lactic acid.

3. Staphylococcus pyogenes aureus in a pulp intensively infected.

The biological properties of the microbes enumerated explain the process of caries. The microbes form lactic acid, and dissolve the mineral substances of the tooth. The microbes disappear in the organic substance and destroy the albuminous bodies. The work of destruction is promoted by the saprogenic microbes present in the mouth.—*Deutsche Medizinal-Zeitung.*

